# HER-2 Receptor and αvβ3 Integrin Dual-Ligand Surface-Functionalized Liposome for Metastatic Breast Cancer Therapy

**DOI:** 10.3390/pharmaceutics16091128

**Published:** 2024-08-27

**Authors:** Dilip Kumar Arya, Hemali Deshpande, Ashish Kumar, Kumarappan Chidambaram, Prashant Pandey, Shabnam Anjum, Payal Deepak, Vikas Kumar, Santosh Kumar, Giriraj Pandey, Saurabh Srivastava, Paruvathanahalli Siddalingam Rajinikanth

**Affiliations:** 1 Department of Pharmaceutical Sciences, Babasaheb Bhimrao Ambedkar University, Lucknow 226025, India; 2 Department of Anatomy, College of Medicine, King Khalid University, Abha 61421, Saudi Arabia; 3 Department of Microbiology and Clinical Parasitology, College of Medicine, King Khalid University, Abha 61421, Saudi Arabia; 4 Department of Pharmacology, College of Pharmacy, King Khalid University, Abha 61421, Saudi Arabia; 5 School of Dentistry, Health Science Center, Shenzhen University, Shenzhen 518015, China; 6 School of Biomedical Engineering, Health Science Center, Shenzhen University, Shenzhen 518015, China; 7 Department of Pharmaceutics, National Institute of Pharmaceutical Education and Research, Hyderabad 500037, India

**Keywords:** Trastuzumab, iRGD, Gefitinib, Lycorine, liposome, HER-2-positive breast cancer

## Abstract

Human epidermal growth factor receptor-2 (HER2)-positive breast cancer metastasis remains the primary cause of mortality among women globally. Targeted therapies have revolutionized treatment efficacy, with Trastuzumab (Trast), a monoclonal antibody, targeting HER2-positive advanced breast cancer. The tumor-homing peptide iRGD enhances the intratumoral accumulation and penetration of therapeutic agents. Liposomes serve as versatile nanocarriers for both hydrophilic and hydrophobic drugs. Gefitinib (GFB) is a potential anticancer drug against HER2-positive breast cancer, while Lycorine hydrochloride (LCH) is a natural compound with anticancer and anti-inflammatory properties. This study developed TPGS-COOH-coated liposomes co-loaded with GFB and LCH, prepared by the solvent injection method, and surface-functionalized with Trast and iRGD. The dual surface-decorated liposomes (DSDLs) were characterized for their particle size (PS), polydispersity index (PDI), zeta potential (ZP), surface chemistry, surface morphology, and their crystallinity during in-vitro drug release, drug encapsulation, and in-vitro cell line studies on SK-BR-3 and MDA-MB-231 breast cancer cells. The half-maximum inhibitory concentration (IC-50) values of single decorated liposomes (SDLs), iRGD-LP, and Trast-LP, as well as DSDLs (iRGD-Trast-LP) on SK-BR-3 cells, were 6.10 ± 0.42, 4.98 ± 0.36, and 4.34 ± 0.32 μg/mL, respectively. Moreover, the IC-50 values of SDLs and DSDLs on MDA-MB-231 cells were 15.12 ± 0.68, 13.09 ± 0.59, and 11.08 ± 0.48 μg/mL, respectively. Cellular uptake studies using confocal laser scanning microscopy (CLSM) showed that iRGD and Trast functionalization significantly enhanced cellular uptake in both cell lines. The wound-healing assay demonstrated a significant reduction in SDL and DSDL-treated MDA-MB-231 cell migration compared to the control. Additionally, the blood compatibility study showed minimal hemolysis (less than 5% RBC lysis), indicating good biocompatibility and biosafety. Overall, these findings suggest that TPGS-COOH-coated, GFB and LCH co-loaded, dual-ligand (iRGD and Trast) functionalized, multifunctional liposomes could be a promising therapeutic strategy for treating HER2-positive metastatic breast cancer.

## 1. Introduction

Metastatic breast cancer remains the leading cause of tumor-related mortality among women globally [1]. While the 5-year survival rate for primary breast cancer patients is approximately 99%, this rate dramatically decreases to around 23% following secondary metastasis [2]. The challenge in treating metastatic breast cancer primarily stems from the inefficient delivery of therapeutic agents, contributing to high mortality rates [3]. Breast cancer metastasizes to multiple organs, notably the brain, lungs, and liver. The metastatic process entails the dissemination of malignant cells from the primary tumor to distant anatomical sites, leading to the establishment of secondary tumors and a substantial rise in mortality rates. Clinically, metastatic breast cancer remains predominantly incurable, with a five-year survival rate of approximately 20% [4]. Despite advancements in current clinical treatments, therapeutic efficacy remains limited, offering only marginal improvements in overall survival. The inefficacy of the existing therapies in treating cancer metastasis is attributed to poor drug delivery efficiency, as many therapeutic agents fail to reach metastatic sites effectively. Nanotherapeutics, leveraging the enhanced permeability and retention (EPR) effect, hold potential for targeting tumors and metastasis [5,6]. However, metastatic sites often consist of small, dispersed clusters of cancer cells in affected organs, posing a significant challenge for effective targeting.

Trastuzumab (Trast), also known as Herceptin, is a humanized monoclonal antibody targeting the extracellular domain of erythroblastic leukemia viral oncogene homolog-2 also known as HER2 (ErbB-2/HER-2), which is overexpressed in 25–30% of breast cancers and their metastases [7]. Despite its targeted approach, clinical response rates to Trast have been limited to 7–35%. One of the major limitations in enhancing treatment efficacy is the absence of an effective drug delivery system capable of facilitating the translocation of therapeutic agents from the cytoplasm to the nucleus. The therapeutic mechanism of Trast involves the reduction in cell proliferation through the induction of DNA double-strand breaks within the nucleus [8,9]. Recent studies have shown that trastuzumab conjugated with a nuclear localization sequence (NLS) can effectively kill Trast-resistant breast cancer cells by inducing DNA damage, underscoring the necessity for developing strategies that enable nuclear delivery for improved therapeutic outcomes [10,11].

The cyclic peptide containing nine amino acids (CRGDKGPDC) referred to as iRGD emerged as a promising tool for tumor-targeted drug delivery and molecular imaging. Its unique structure integrates functionalities for both specific targeting and enhanced penetration into the tumor cells [12,13]. iRGD exhibits a high affinity for αvβ3 and αvβ5 integrins, which are overexpressed on tumor neovasculature and certain tumor cells [14]. This facilitates tumor homing and the accumulation of iRGD-conjugated therapeutic agents or co-administered drugs. Notably, iRGD possesses a cryptic CendR motif (RGDK) that becomes exposed upon proteolytic cleavage within the tumor microenvironment. This exposed motif binds to neuropilin-1 (NRP-1), a receptor upregulated in aggressive tumors, promoting the deeper penetration of iRGD and its cargo into the extravascular tumor parenchyma. By exploiting both integrin-mediated targeting and NRP-1-facilitated penetration, iRGD offers a novel strategy for efficient tumor theragnostic [15,16]. Lycorine hydrochloride (LCH), a derivative of the isoquinoline alkaloid lycorine extracted from Lycoris plants, has emerged as a promising candidate for cancer therapy [17,18]. Prior studies have established the pleiotropic pharmacological properties of LCH, encompassing antitumor, antiviral, anti-inflammatory, antimalarial, and acetylcholinesterase inhibitory activities [19]. Notably, LCH and its derivatives have demonstrated significant inhibitory effects on a spectrum of malignancies, including leukemia, lymphoma, melanoma, esophageal cancer, breast cancer, ovarian cancer, and prostate cancer [20,21]. It is also known to inhibit tumor neovascularization. Intriguingly, existing evidence suggests a preferential cytotoxic effect of LCH on tumor cells compared to normal cells. However, the specific effects of LCH on breast cancer remain unexplored. Therefore, a comprehensive investigation into the efficacy of LCH against breast cancer and an elucidation of the underlying mechanisms of action is warranted [22,23]. Previous research has demonstrated that LCH inhibits cell proliferation in ovarian carcinoma and osteosarcoma by inducing cell cycle arrest. Additionally, LCH facilitates autophagy and apoptosis in hepatocellular carcinoma cells through the inactivation of the tongue cancer resistance-associated protein (TCRP1)/Protein kinase B (Akt)/mammalian target of rapamycin (mTOR) (TCRP1/Akt/mTOR) signaling pathway. In a breast cancer mice model, LCH could potently inhibit tumor growth and pulmonary metastasis without apparent toxicity. Additionally, LCH dramatically enhanced the tumor-suppressive effect [19].

Gefitinib (GFB), a highly lipophilic drug (BCS Class II, log P = 3.2, pKa 5.28, 7.17), exhibits slow absorption and limited oral bioavailability due to pH-dependent dissolution and potential efflux mechanisms [24]. GFB is a first-generation tyrosine kinase inhibitor (TKI), reversibly inhibiting EGFR autophosphorylation and downstream mitogen-activated protein kinase/phosphatidylinositol 3-kinase (MAPK/PI3K) signaling in EGFR-overexpressing cancers (breast, lung, colon, brain). Its widespread distribution to EGFR-expressing tissues necessitates a targeted delivery system to optimize therapeutic efficacy and minimize adverse effects (rash, diarrhea, lung disease, cardiotoxicity). Nanoparticle-based delivery methods may enhance therapy for HER-2-positive breast cancer by modulating immune response and improving tumor penetration by sustaining the release of GFB and LCH. More effective drug delivery strategies are crucial to potentially reduce dosing, enhance solubility, and reduce adverse effects [25,26,27].

D-Alpha-Tocopheryl polyethylene glycol 1000 succinate (TPGS) is a vitamin E derivative that is soluble in water with improved pharmaceutical properties. This amphiphilic molecule with a hydrophilic and lipophilic balance (HLB) value between 15 and 19 functions as an effective surfactant due to its balanced hydrophilic and lipophilic domains [28,29]. Co-administration of TPGS enhances solubility, inhibits P-glycoprotein-mediated multi-drug resistance, and improves the oral bioavailability of chemotherapeutic agents [30,31]. It serves as a versatile tool in chemotherapeutic delivery via nanoparticles, acting as an emulsifier, stabilizer, bioavailability enhancer, solubilizer, and P-glycoprotein inhibitor [32,33]. FDA-approved TPGS demonstrates improved drug encapsulation efficiency, cellular uptake, in-vitro cytotoxicity, and prolonged nanoparticle circulation. TPGS-coated liposomes exhibit significantly higher cytotoxicity and lower IC-50 compared to 1,2-distearoyl-sn-glycero-3-phosphoethanolamine-N-[amino(polyethylene glycol)-2000] (DSPE-PEG2000) counterparts C6 glioma cells [34]. On the liposomal surface, carboxyl-terminated TPGS, or activated TPGS (TPGS-COOH), is used to enhance covalent conjugation [35]. Lipid-based nanoparticles, known as liposomes, have been widely used in systemic drug delivery applications [36,37]. Currently, a variety of liposomal formulations are used in clinical settings to improve the delivery of siRNAs and anticancer medications. Liposomes have the capability to encapsulate both lipid-soluble and water-soluble drugs within their lipid bilayers. This encapsulation of drugs within liposomes effectively shields the negative charges of nucleotides and protects them from nuclease degradation. Additionally, it prevents the rapid degradation of drugs encapsulated within their structure and reduces their systemic toxicity by limiting their availability in the bloodstream [38,39]. The prolonged plasma half-life of the therapeutic substances encapsulated in liposomes is also a benefit that results from their reduced size [40,41,42].

**Scheme 1:**
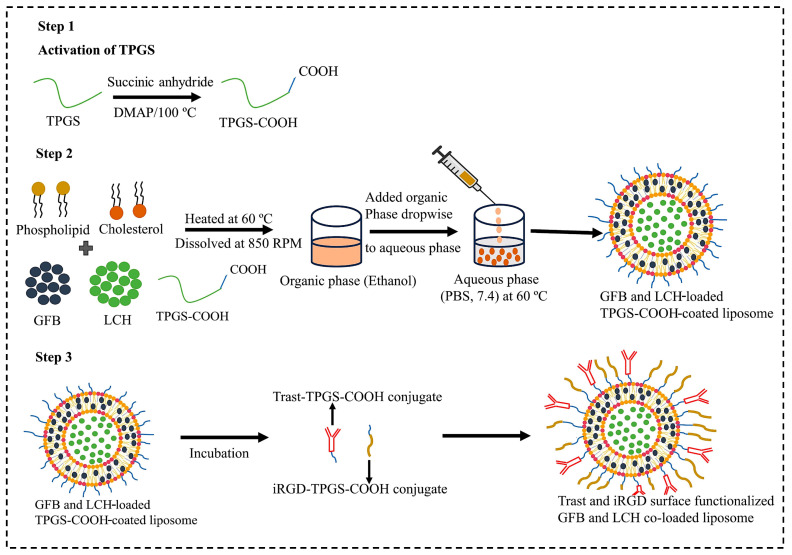
Schematic representation of preparation of surface-decorated liposome by solvent injection method and functionalization with Trast monoclonal antibody and iRGD (tumor-penetrating ligand) and loaded with GFB and LCH anticancer drugs.

Extravascular targets within the TME play crucial roles in tumor growth, progression, as well as metastasis, and their modulation can significantly improve therapeutic outcomes. Key targets include cancer-associated fibroblasts (CAFs), which promote tumor proliferation, invasion, and resistance via the secretion of growth factors, cytokines, and ECM components. The ECM itself impedes drug delivery, but targeting elements like collagen and hyaluronan can enhance therapeutic efficacy [43]. Tumor-associated macrophages (TAMs) and myeloid-derived suppressor cells (MDSCs) further contribute to immune evasion and tumor progression [44]. Signaling molecules within the TME, including transforming growth factor beta (TGF-β), vascular endothelial growth factor (VEGF), and interleukins, facilitate tumor growth, angiogenesis, and immune suppression, with their inhibition offering a route to disrupt these oncogenic pathways. Tumor-infiltrating lymphocytes (TILs), depending on their phenotype, may either support or combat tumor growth; thus, modulating their activity can strengthen anti-tumor immunity. Additionally, tumor hypoxia and the activation of hypoxia-inducible factors (HIFs) promote angiogenesis and metabolic reprogramming, with HIF inhibition offering a potential therapeutic strategy. Current and emerging therapies in HER-2-positive breast cancer not only target HER-2 receptors but also extend to HER3, programmed-death ligand (PD-L1), cytotoxic T-lymphocyte-associated protein-4 (CTLA-4), natural killer group 2A (NKG-2A), AKT, PI3K, estrogen receptors, and cyclin-dependent kinases 4/6 in triple-positive tumors [45]. In order to improve the selectivity and accessibility of therapeutics to extravascular targets and tumor tissues, we covalently attached Trast and the tumor-penetrating peptide iRGD onto the surface of liposomes co-loaded with GFB and LCH using TPGS-COOH as depicted in Scheme 1. This peptide can carry conjugated cargo, such as small molecules and nanoparticles, across the tumor vasculature and deep into the extravascular tumor matrix. It also makes it easier to selectively target a variety of solid tumors when administered systemically. Additionally, this vascular and tumor-penetration capability extends to co-administered therapeutics not directly linked to the peptide, known as the bystander effect. The multifunctional iRGD-Trast functionalized liposomes, loaded with both GFB and LCH, are proposed to significantly enhance drug delivery efficiency into various solid tumors, particularly in targeting HER2-positive breast cancer brain metastases.

## 2. Materials and Methods

### 2.1. Materials

GFB were generously provided by Heterodrugs Ltd., Hyderabad, India. LCH, lipoid E-80 (E-80), TPGS, Cholesterol, 1-Ethyl-3-[3-dimethylaminopropyl] carbodiimide hydrochloride (EDC), sulfo-N-hydroxy succinimide (sulfo-NHS), 4-(Dimethyl amino) pyridine (DMAP), and Triton X-100 were procured from Sigma-Aldrich (St. Louis, MO, USA). Trast and iRGD were obtained from APExBIO Technology (Houston, TX, USA). Additionally, 3-(4,5-dimethylthiazol-2-yl)-2,5-diphenyltetrazolium bromide (MTT), 4′,6-diamidino-2-phenylindole dihydrochloride (DAPI), and coumarin-6 were also acquired from Sigma-Aldrich, St. Louis, MO, USA. Dulbecco’s Modified Eagle Medium (DMEM), trypsin-EDTA (0.25%), and fetal bovine serum (FBS) were sourced from Himedia, Mumbai. Dialysis membranes with a molecular weight cutoff between 12,500 and 14,000 daltons were purchased from Himedia, Mumbai. A 0.2 µm syringe filter was obtained from Thermo Fisher Scientific Pvt. Ltd., Maharashtra, India. All other chemicals and reagents utilized were of analytical grade. The SK-BR-3 and MDA-MB-231 cell lines were acquired from the National Centre for Cell Science (NCCS), Pune, India.

### 2.2. Methods

#### 2.2.1. Synthesis of TPGS-COOH

TPGS-COOH was synthesized using a ring-opening polymerization mechanism. TPGS (0.77 gm) and succinic anhydride (0.10 gm) along with DMAP (0.12 gm) were mixed. The mixture was then allowed to react at 100 °C under nitrogen for 24 h. Then, 5 mL of cold dichloromethane (DCM) was used to collect the reaction mixture once it had cooled to room temperature and filtered to remove excess succinic anhydride. The filtrate was then allowed to precipitate overnight at −10 °C in 100 mL of diethyl ether. The white precipitate was further filtered and vacuum-dried in order to obtain TPGS-COOH.

#### 2.2.2. Synthesis of Trast-TPGS-COOH Hybrid Conjugate

Phosphate-buffered saline (PBS, pH 5.5) was used to dilute the TPGS-COOH-based liposomes (0.5 mL, 2.5 mg/mL TPGS-COOH) and incubated with 200 µL of 100 mM EDC and 200 µL of 25 mM NHS for 30 min at normal temperature with gentle stirring (to activate the carboxyl groups on the liposomes). The activated particles were then incubated with 0.5 mL of Trast solution (5 µg/mL) for another 30 min at room temperature for antibody conjugation. The antibody–liposome conjugates were further dialyzed using a 1000 Da dialysis membrane for 4 h to obtain the purified conjugate.

#### 2.2.3. iRGD-TPGS-COOH Hybrid Conjugate

iRGD peptide conjugation to TPGS-COOH was performed by carbodiimide chemistry, which involves the formation of an amide bond (-CO-NH) between the amine group (-NH_2_) of iRGD and the carboxylic group (-COOH) of TPGS-COOH, respectively. TPGS-COOH was incubated with EDC and NHS (as a catalyst) at a 1:5 molar ratio to obtain the iRGD-TPGS-COOH conjugate. TPGS-COOH (200 mg), EDC (96 mg), and NHS (74 mg) were dissolved in 1 mL of PBS at pH 7.4 at 25 °C and incubated for 5 h, followed by storage at 4 °C for 24 h. Finally, 500 µL of 0.1% (*w*/*v*) iRGD was mixed with the previously activated TPGS-COOH and incubated at 4 °C for 8 h with continuous stirring at 100 rpm. The iRGD-TPGS-COOH conjugate was further purified by conducting dialysis with the help of a dialysis membrane (MWCO: 1 kDa) against PBS (pH 7.4) for 5 h and collected by freeze drying [46].

#### 2.2.4. Experimental Design and Optimization of GFB and LCH Dual Drug-Loaded Liposome

Response surface methodology (RSM) was utilized as a design of experiments (DOE) approach to formulate and optimize drug-loaded liposomes. A total of 15 experimental trials were executed. The optimization was carried out using Design-Expert^®^ software (Version 13.0, Stat-Ease Inc., Minneapolis, MN, USA). A Box–Behnken Design (BBD) with 3 factors and 3 levels was employed to evaluate the effects of the independent variables E-80 (X1), Cholesterol (X2), and TPGS (X3) on the dependent variables, which included particle size (PS) (Y1), polydispersity index (PDI) (Y2), and zeta potential (ZP) (Y3). The sonication time was fixed at 10 min. Three levels (−1, 0, and +1) were used to vary the independent variables, meaning that the values were low, medium, and high, respectively (Table 1).

The parameters of this model were then estimated using standard fitting techniques. The goodness of fit was measured by the correlation coefficient (R^2^) value, which provided a measure of how well the model explained the variability in the response variable. A higher R^2^ indicates a better fit. Additionally, the adjusted R^2^ value was measured to account for the number of predictors in the model, providing a more accurate assessment of model performance in the context of multiple predictors. The root mean square error (RMSE) was also used to quantify the differences between observed and predicted values. Model diagnostics were assessed to ensure the validity and reliability of the model. Thus, the software identified the optimal combination of the independent variables that resulted in the desired characteristics of the liposomes, such as smaller PS, lower PDI, and appropriate ZP values. Additionally, the effects of different factors on the response values were analyzed using Design-Expert^®^ software. This analysis involved generating various 3D response surface plots and 2D contour plots. These graphical representations allowed for the visualization of the interactions between the independent variables and their combined impact on the response variables, providing a comprehensive understanding of the optimization process.

#### 2.2.5. Surface Functionalization of Optimized Liposome by iRGD and Trast

After selecting the optimized formulation, surface-decorated, dual-drug-loaded liposomes (DSDLs) were prepared using the solvent injection method. Briefly, specific amounts of GFB (10 mg), LCH (1 mg), E-80 (44.07 mg), cholesterol (19.2 mg), and TPGS-COOH (5.5 mg) were dissolved in 1 mL of ethanol at 60 °C. This mixture was then injected isothermally at 60 °C into 5 mL of PBS (pH 7.4). The resulting suspension was maintained at 60 °C with stirring for 1 h at 850 rpm followed by probe sonication (Labman, Model-Pro-650). To ensure lipid remained above the phase transition temperature (Tm) during filtration, the prepared liposomes were passed through a 0.22 µm syringe filter. Finally, the liposomes were centrifuged at 11,000 rpm for 15 min to remove excess non-incorporated drugs. These liposomes were then stored at 4 °C for further use. Subsequently, for surface decoration via hydrophobic interaction, either Trast-TPGS-COOH hybrid, iRGD-TPGS-COOH hybrid, or both were added to the liposomes and incubated for 1 h in a water bath shaker. After 1 h, the DSDLs were dialyzed using dialysis membrane for 2–3 h to remove the non-conjugated liposomes.

## 3. Characterization of DSDLs

### 3.1. Zeta Analysis

PS, ZP, and PDI were determined by dynamic light scattering using Zetasizer Model ZEN3690 Ver. 7.03. Serial measured parameters were average PS diameter (ZAve), PDI, and ZP (±mV), reflecting the mean diameter of the particles, their size distribution, and their stability [47,48].

### 3.2. Scanning Electron Microscopy (SEM) Analysis

The size and shape of the liposomal formulation were confirmed by SEM analysis (Model: JSM 6490 LV, JEOL, Akishima, Japan). One drop of the liposome was spin-coated on a glass slide at 1500 rpm for 60 s under vacuum, then fixed on an aluminum stub using carbon tape, and coated with platinum using sputter coating. Finally, images were taken at different magnifications and voltages. SEM offers high-resolution imaging capabilities that make it possible to characterize liposome morphology. Understanding liposome physical characteristics and structure is crucial since these factors can affect their stability, drug encapsulation, and interactions with biological systems [49,50,51,52].

### 3.3. Transmission Electron Microscopy (TEM) Study

The transmission electron microscopy (TEM) system (Philips CM-12, Fullerton, CA, USA) was used to examine the liposome’s surface morphology. One drop of diluted liposome was placed on a copper grid and cured under vacuum pressure for 1 min. After 30 s of 1% phosphotungstic acid staining, the dried liposomes were evaluated [53,54].

### 3.4. X-ray Diffraction (XRD) Study

X-ray diffraction (XRD) analysis was conducted to evaluate the purity and crystalline structure of the drugs and DSDL formulations utilizing a D8 Advance Eco, X-ray diffractometer (Bruker, Karlsruhe, Germany) equipped with Cu Kα radiation, operating at parameters of 30 kV and 15 mA. The measurements were performed at ambient temperature, spanning a 2θ diffraction angle range of 0–80°, with a scanning speed of 1° per minute [55,56].

### 3.5. Fourier Transform Infrared Spectrophotometer (FTIR) Analysis

The presence of functional groups in the chemicals and their conjugation to biocompatible formulations was confirmed by Fourier transform infrared spectroscopy (FTIR) analysis using a Thermo-Scientific Nicolet 6700 instrument. The FTIR spectra covered a wide range, capturing peaks from 4000 to 400 cm^−1^ [57].

### 3.6. Determination of Encapsulation Efficiency (EE%) and Drug Loading (DL%)

The encapsulation efficiency (EE%) of DSDLs was determined by reversed-phase high-performance liquid chromatography (RP-HPLC) (Shimadzu, Prominence-I, LC-2030 plus) using a centrifugal device (Nanosep with 30 K, Pall Corporation) having a pore size below 5 nm. Then, 500 µL of DSDL was added into the Nanosep tube and centrifuged at 11,000 rpm for 15 min. After 15 min of centrifugation, the filtrate containing DSDLs was collected and dissolved in mobile phase. Next, it was filtered by using a 0.22 µm syringe filter and placed in an RP-HPLC vial for analysis. The concentration of the encapsulated drug was measured using previously developed RP-HPLC [58,59,60,61]. The following formula was used to determine the GFB and LCH encapsulation efficiencies.

$$DL\;\left(\%\right)=\frac{Weight\;of\;drug\;present\;in\;liposome}{Total\;weight\;of\;liposome}\times 100$$

$$EE\;\left(\%\right)=\frac{Amount\;of\;drug\;present\;in\;liposomes}{Amount\;of\;drug\;added\;during\;fabrication}\times 100$$

### 3.7. Stability Studies

The developed DSDL formulation was subjected to stability study as per the International Council for Harmonization of Technical Requirements for Pharmaceuticals for Human Use (ICH) guidelines. Sampling was made at 0 days, 1 day, 7 days, 15 days, 1 month, 3 months, and 6 months [62,63,64].

### 3.8. In-Vitro Drug Release Study

The release kinetics of GFB and LCH from DSDLs were examined using the dialysis bag diffusion method. The DSDL was placed into a dialysis bag made of a cellulose membrane with a molecular weight cutoff of 12 kDa. The dialysis bag was then submerged in 20 mL of PBS at a pH of 7.4 and kept at a consistent temperature of 37 ± 0.5 °C. The release medium, which contained 1% Tween 80, was constantly agitated at 100 rpm. Aliquots were taken out of the receptor compartment and promptly replaced with an equivalent volume of fresh release media at predetermined intervals (0, 0.5, 1, 2, 4, 6, 8, 10, 12, 24, and 48 h). Before analysis, a 0.22 µm syringe filter was used to filter the samples. GFB was quantified using an RP-HPLC technique that had been previously verified using a C18 column. Acetonitrile (ACN), methanol, and water were combined in the mobile phase at a ratio of 70:20:10, with a flow rate of 1 mL/min, and a detection wavelength of 254 nm. Moreover, a C18 column with a mobile phase consisting of 0.01% trifluoroacetic acid (TFA) and ACN at a 90:10 ratio was used to quantify LCH at 290 nm [65,66,67].

### 3.9. Blood Compatibility Study

In-vitro hemocompatibility is a crucial parameter for any developed nanoformulation. Different developed liposomal formulations were tested for any hemolytic effect. Blood samples were withdrawn from rats and collected in heparin tubes. The supernatant was extracted after centrifuging the blood for 10 min at 5000 rpm and 4 °C. The red blood cells (RBCs) pellet was subjected to three consecutive wash cycles with PBS (pH 7.4). The pellets were re-dispersed in 5 mL of PBS (pH 7.4) after each wash, and then the mixture was centrifuged at 3000 rpm for 10 min at 4 °C. Finally, the RBCs pellet was diluted in 25 mL of PBS (pH 7.4) to perform the hemolysis study. Distilled water was used as a positive control (complete lysis) and PBS (pH 7.4) as a negative control (no lysis). Previously diluted RBCs suspension (1 mL) was added to a 1.5 mL Eppendorf tube, and 0.5 mL of liposomal formulation (SDL and DSDL at a concentration of 20 µg/mL) was added and incubated at 37 °C for 3 h. To determine the amount of hemolysis, each tube was centrifuged for 5 min at 4 °C at 5000 rpm following incubation. After collecting the supernatant, the percentage of RBCs destruction was determined by measuring the absorbance of the supernatant at 540 nm using an ELISA plate reader. The following formula was utilized to calculate the percentage of hemolysis of all the samples [68,69,70]:

$$Hemolysis\;\left(\%\right)=\frac{Absorbance_{sample}-Absorbance_{negative\;control}}{Absorbance_{positive\;control}-Absorbance_{negative\;control}}$$

### 3.10. In-Vitro Cell Study

#### 3.10.1. In-Vitro Cytotoxicity (MTT Assay)

The cytotoxicity of SDLs and DSDLs was evaluated on SK-BR-3 and MDA-MB-231 metastatic breast cancer cell lines obtained from NCCS, Pune, India. The MTT assay, a colorimetric method, measures the activity of viable cells based on their ability to convert MTT dye into a colored product (insoluble formazan crystal, purple color). Briefly, SK-BR-3 and MDA-MB-231 cells were cultured in 96-well plates at a density of 1 × 10^4^ cells/well in DMEM supplemented with 1% (*v*/*v*) antibiotic solution and 10% (*v*/*v*) FBS. Cells were incubated at 37 °C with 5% CO_2_ for 24 h. The SDL and DSDL formulations were then applied at concentrations ranging from 1 to 10 μg/mL for SK-BR-3 and 1–25 μg/mL for MDA-MB-231, respectively, and incubated for another 24 h to check the cytotoxic potential of the developed SDL formulations. Following incubation, each well was treated with 20 μL of a 5 mg/mL MTT solution for 4 h. This allowed viable cells to convert MTT reagents into purple formazan crystals by dehydrogenases and reductases present in the mitochondria of metabolically active cells. To dissolve these crystals for measurement, 100 μL of dimethyl sulfoxide (DMSO) was added to each well and mixed thoroughly with a plate shaker. The IC_50_ values of SDL and DSDL formulations were calculated based on the absorbance measured at 570 nm using an ELISA plate reader [71,72,73].

#### 3.10.2. Cellular Uptake by Confocal Laser Scanning Microscopy (CLSM)

For CLSM, cells were seeded in a 12-well plate containing a glass coverslip in each well at an initial density of 2 × 10^5^ cells/well and incubated overnight. Coumarin-6-loaded placebo SDLs and DSDLs were added to cells and incubated for 2 h at 37 ± 0.5 °C in an incubator with 5% CO_2_. After 2 h, the SDL and DSDL formulations were removed and cells were rinsed three times with PBS (pH 7.4) and fixed with 1 mL of 4% paraformaldehyde solution for 30 min and finally treated with 1 mL of 0.1% Triton X-100 for 5 min to enhance the permeability of DAPI. Then, the nuclei were stained with DAPI (5 µg/mL) for 10 min. After staining, the cells were washed 3 times with PBS (pH 7.4). Glass coverslips were further removed from the 12-well plate and mounted on a glass slide. Subsequently, cellular internalization was examined in cells using a CLSM (Model-LSM900, Zeiss, Germany) at a magnification of 20×.

#### 3.10.3. Wound-Healing Assay

The 12-well plate containing 1 × 10^5^ cells/well of cultivated MDA-MB-231 cells was seeded and incubated at 37 °C for 24 h to reach 70% confluency. The scratch was created using a 200 µL pipette tip, and the debris was rinsed out three times with PBS (pH 7.4). The SDL (iRGD-LP and Trast-LP) and DSDL (iRGD-Trast-LP) formulations were added into the 12-well plate at a concentration of half of their corresponding IC_50_ values. Then, fresh 2 mL of complete media was added to each well. The cells were incubated at 37 °C for 24 h to measure the wound-healing capacity of SDL and DSDL-treated MDA-MB-231 cells against the control group. To evaluate the possibility of cellular migration, the samples were examined under an optical microscope at 0 and 24 h [74,75,76].

### 3.11. Statistical Analysis

The data, derived from a minimum of three independent experiments, are presented as mean ± S.D. Student’s *t*-test was utilized for statistical comparisons between two groups, and one-way analysis of variance (ANOVA) was applied for comparisons between multiple groups, followed by Tukey’s post hoc test. All experimental analyses were conducted using GraphPad Prism version 8 software.

## 4. Results

### 4.1. Experimental Design and Optimization of Liposomes

#### 4.1.1. BBD 3 Factor-3 Level Approach

The GFB and LCH-loaded liposomes were successfully prepared by the solvent injection method and optimized using BBD by applying design expert software 13. RSM was employed to analyze the effect of independent variables (E-80, cholesterol, and TPGS) on dependent variables (PS, PDI, and ZP). In total, 15 experiment runs were performed for this investigation and their results are presented in Table 2.

##### Effect of Formulation Parameters (E-80, Cholesterol, and TPGS) on PS

**Figure 1:**
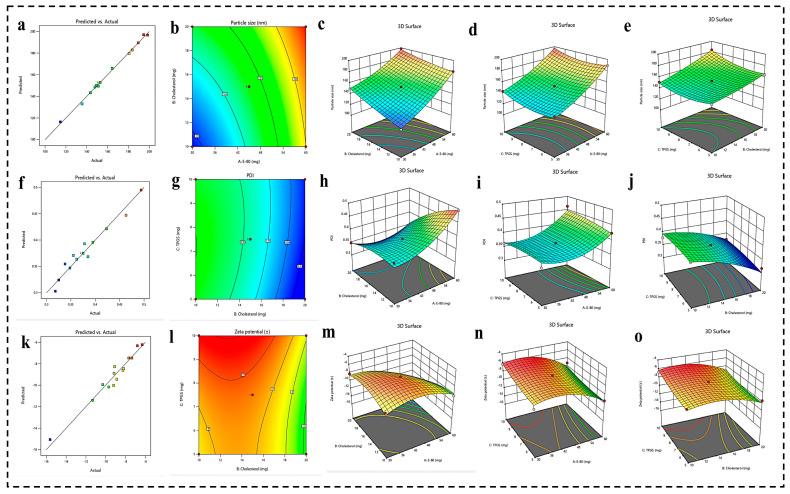
(**a**–**e**) Prediction versus actual graph, 2D contour plot, and 3D response surface graph representing the impact of different independent variables (E-80, cholesterol, and TPGS) on PS. (**f**–**j**) Prediction versus actual graph, 2D contour plot, and 3D response surface graph representing the impact of different independent variables (E-80, cholesterol, and TPGS) on PDI. (**k**–**o**) Prediction versus actual graph, 2D contour plot, and 3D response surface graph representing the impact of different independent variables (E-80, cholesterol, and TPGS) on ZP.

PS represents a pivotal parameter in nanoformulation, significantly influencing both cellular internalization and biodistribution. The relevance of the quadratic model employed for the developed formulations was assessed through an examination of the F-value and *p*-value. The observed F-value of 60.41 and *p*-value of 0.0034 indicate that the quadratic model is highly statistically significant, meaning it explains the relationship between the factors and the responses well. Additionally, the ANOVA results reveal an R^2^ value of 0.9969, demonstrating an optimal model fit. The discrepancy between the adjusted R^2^ and predicted R^2^ was found to be less than 0.2. The impact of various factors on PS was evaluated via their respective *p*-values. The *p*-values for E-80, cholesterol, and TPGS were 0.0001, 0.0001, and 0.0024, respectively, all of which are below the threshold of 0.05, indicating that these factors exert a statistically significant influence on PS. Figure 1a–e illustrates the effect of each independent variable on PS through 3D response surface graphs. The RSM generated the following polynomial regression equation for PS as a function of A (E-80), B (cholesterol), and C (TPGS).

PS=+152.8+27.5125 ∗A+12.65 ∗ B+4.46 ∗ C−4.17 ∗ AB−0.7000 ∗ AC+4.03 ∗ BC+5.98 ∗ A2+1.95 ∗ B2+6.97 ∗ C2

where A, B, and C represent E-80, cholesterol, and TPGS concentration, respectively.

##### Effect of Formulation Parameters (E-80, Cholesterol, and TPGS) on PDI

PDI is a critical parameter reflecting the uniformity of particle size distribution within nanoformulation. A PDI value below 0.5 denotes a narrow size distribution, indicating that the majority of particles exhibit homogeneity in size. The analysis yielded an F-value of 5.50 and a *p*-value of 0.048, signifying that the model is statistically significant and adequately elucidates the relationship between the independent factors and the response variable. The ANOVA results demonstrate an R^2^ value of 0.9536, indicating that the model reliably assesses the impact of the examined factors on the response variable. Further evaluation of the influence of various factors on the PDI was conducted through their respective *p*-values. The *p*-values for E-80, cholesterol, and TPGS were 0.0028, 0.0012, and 0.4862, respectively. These results suggest that both E-80 and cholesterol exert a statistically significant effect on PDI, as indicated by their low *p*-values. However, the *p*-value of 0.4862 for TPGS suggests that its concentration does not significantly influence the PDI value. Figure 1f–j presents the effects of each independent variable on PDI through 3D response surface graphs. The RSM generated the following polynomial regression equation, delineating PDI as a function of A (E-80), B (cholesterol), and C (TPGS):

PDI=+0.375+0.0335 ∗ A− 0.0404 ∗ B+0.0046 ∗ C−0.0297 ∗ AB+0.0083 ∗ AC+0.006 ∗ BC+0.0315 ∗ A2−0.0152 ∗ B2−0.00575 ∗ C2

where A, B, and C represent E-80, cholesterol, and TPGS concentration, respectively.

##### Effect of Formulation Parameters (E-80, Cholesterol, and TPGS) on ZP

The ZP in nanoformulation, in particular liposomes, plays a pivotal role as it directly affects their stability, biodistribution, drug delivery efficiency, and surface-modification capabilities. A higher absolute ZP value ensures better electrostatic repulsion between liposomes, preventing aggregation and prolonging the shelf life of liposomes. It also influences encapsulation efficiency, cellular uptake, and interaction with biological components, thereby enhancing circulation time and targeting capabilities. Moreover, the ZP plays a significant role in the successful attachment of functional molecules and polymers to the liposome surface, enhancing biocompatibility and reducing immune recognition. The analysis resulted in an F-value of 11.69 and a *p*-value of 0.0107, indicating that the model is highly statistically significant and effectively elucidates the relationship between the independent variables and the response variable. The ANOVA results reveal an R^2^ value of 0.9589, signifying that the model robustly captures the influence of the studied factors on ZP. Further investigation into the impact of each factor on the PDI was conducted via their respective *p*-values. The *p*-values for E-80, cholesterol, and TPGS were 0.0014, 0.0110, and 0.0256, respectively, suggesting that E-80 has the highest pronounced effect on ZP compared to cholesterol and TPGS. Nonetheless, cholesterol and TPGS also exert statistically significant effects on PDI, as evidenced by their *p*-values. Figure 1k–o illustrates the influence of each independent variable on PDI through 3D response surface graphs. The RSM generated the following polynomial regression equation, describing ZP as a function of A (E-80), B (cholesterol), and C (TPGS):

ZP=−7.463−1.74 ∗ A−1.07 ∗ B+0.8538 ∗ C−1.49 ∗ AB−0.1675 ∗ AC−0.215 ∗ BC−1.91 ∗ A2−1.39 ∗ B2+0.3928 ∗ C2

where A, B, and C represent E-80, cholesterol, and TPGS concentration, respectively.

#### 4.1.2. Optimization of Formulation by Numerical Analysis and Point Prediction

**Figure 2:**
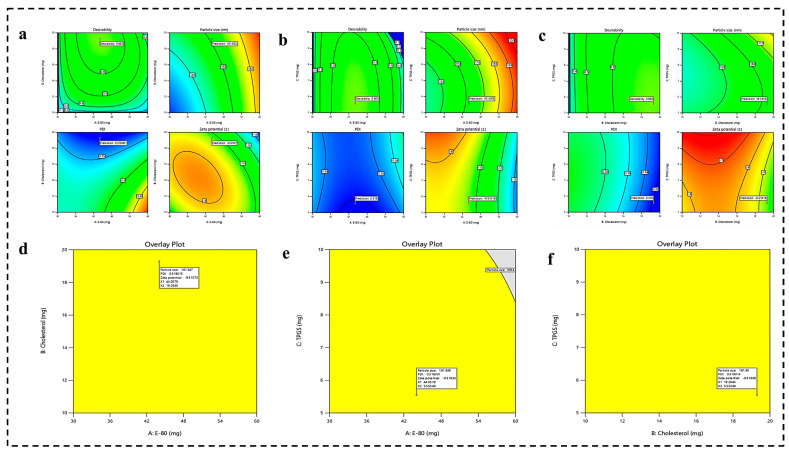
(**a**–**c**) The desirability graph representing the effect of independent (E-80, cholesterol, and TPGS) on dependent variables (PS, PDI, and ZP) and predicting the actual responses for PS, PDI, and ZP at desired level of independent variables. (**d**–**f**) Optimization of dependent variables (PS, PDI, and ZP) using overlay plot.

After analyzing the impact of each factor on responses, the final optimized formulation was obtained by the numerical and graphical optimization technique, as illustrated in Figure 2. Numerical optimization makes it possible to decide the desired level of independent and dependent variables. E-80 and cholesterol were fixed at the maximum, whereas TPGS was set within the range. The PS and PDI responses were set at the minimum level, whereas ZP was set at the maximum level. Finally, point prediction was used to estimate the responses at the optimized formulation parameters, and confirmatory experiments were conducted to validate the predicted results. This comprehensive approach ensures a robust and efficient formulation with desired characteristics. The predicted values for PS, PDI, and ZP were 161.67 nm, 0.318, and −9.51 mV, respectively.

### 4.2. Zeta Analysis

**Figure 3:**
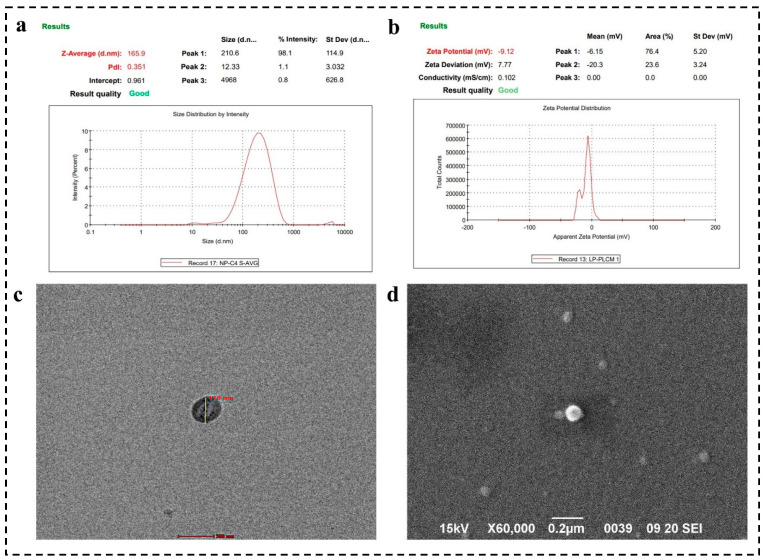
(**a**) The average PS and PDI of optimized formulation, (**b**) ZP analysis, (**c**) TEM images confirmed the spherical shape of liposomes and size within 200 nm, (**c**,**d**) SEM photographs represented the spherical morphology of surface-camouflaged liposomes developed by using solvent injection method.

The PS, PDI, and ZP are essential parameters influencing the stability and colloidal behavior of liposomes. Our study reveals that liposomes possess an average hydrodynamic diameter of 165.9 ± 3.03 nm and a PDI of 0.359 ± 0.034, indicating a uniform size distribution, as depicted in Figure 3a. The ZP was −9.12 ± 0.038 mV, reflecting a net negative surface charge, likely due to the ionization of phosphatidylcholine groups, as evidenced in Figure 3b. This negative charge enhances the electrostatic repulsion between liposomes, enhancing colloidal stability and preventing aggregation. The nanoscale size (50–200 nm) of these liposomes promotes cellular uptake and bioavailability. The low PDI value (*p* < 0.5) suggests a narrow size distribution, which contributes to the long-term stability of the liposomal formulation. The high negative ZP correlates with increased stability in colloidal systems, predicting minimal aggregation and robust dispersion during storage.

### 4.3. Surface Morphology by SEM and TEM Analysis

SEM analysis confirmed the uniform, spherical morphology of the liposomes, a crucial characteristic for drug delivery applications, as shown in Figure 3d. This morphology enhances cellular uptake due to the increased contact area with target cells, ultimately improving therapeutic efficacy. SEM analysis of the synthesized liposomes revealed a size range between 100 and 200 nm. SEM images demonstrated that the liposomes maintained a spherical shape, which is typical for these vesicular structures. The uniformity in shape and size distribution suggests a consistent preparation method, crucial for application in HER-2-positive breast cancer targeting. The consistent PS of liposomes can influence circulation time, cellular uptake, and distribution within the body. This size of nanocarrier facilitates the EPR effect, which allows for better accumulation of liposomes in tumor tissues due to their leaky vasculature. The size also supports the encapsulation of a substantial volume of therapeutic agents (GFB and LCH) while still allowing the liposomes to penetrate tissues efficiently.

Moreover, the spherical morphology observed in TEM ensures a uniform surface area for potential functionalization with targeting ligands (iRGD and Trast), enhancing specificity and efficacy, as mentioned in Figure 3c. The relatively narrow size distribution (100–200 nm) allows for more precise functionalization strategies. Functional groups or targeting ligands can be uniformly distributed across the liposome surface, enhancing targeted delivery capabilities. This precision in functionalization is particularly important in developing targeted therapies for HER-2 positive breast cancer, where selective binding to specific cell types can significantly improve therapeutic outcomes. Moreover, uniformly sized and shaped liposomes, as observed, can efficiently release the therapeutic agents, ensuring sustained and controlled release at the target site.

### 4.4. Characterization of Hybrid Conjugates FTIR Analysis

#### 4.4.1. Characterization of TPGS-COOH by FTIR

**Figure 4:**
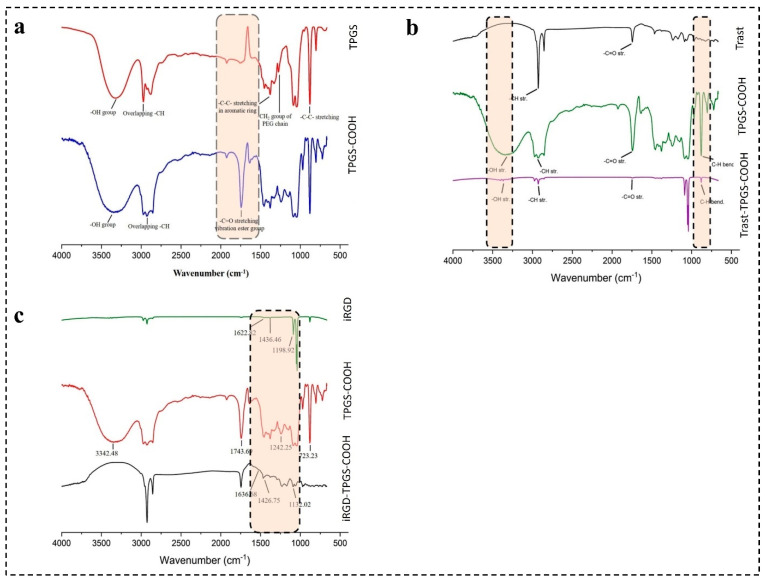
FTIR spectra of (**a**) TPGS and TPGS-COOH; (**b**) Trast, TPGS-COOH, and Trast-TPGS-COOH; and (**c**) iRGD, TPGS-COOH, and iRGD-TPGS-COOH.

TPGS was successfully activated to TPGS-COOH by the ring-opening polymerization mechanism and characterized by FTIR spectra, as seen in Figure 4a. The FTIR spectra of TPGS reflected the main characteristic peaks at 2870.45, 1453.06, and 1379.94 cm^−1^. The presence of a broad absorption peak at 2870.45 cm^−1^ is associated with O-H stretching vibrations, confirming the presence of a hydroxyl group within the TPGS molecule. Moreover, the peaks observed at 1453.06 cm^−1^ and 1379.94 cm^−1^ are associated with the presence of -C-C-stretching vibrations in the aromatic ring of TPGS and C-O stretching vibrations in the PEG chain in the ether linkage (C-O-C) present in TPGS.

Furthermore, the sharp peak observed around 3000 cm^−1^ was due to the C-H stretching vibrations from methylene (CH_2_) in PEG chain and methyl (CH_3_) groups present in TPGS.

The FTIR spectrum of TPGS-COOH displayed characteristic peaks at 1743.69 cm^−1^, 2930.67 cm^−1^, and a broad peak around 3300–3500 cm^−1^. The peak at 1743.69 cm^−1^ indicates the presence of a carbonyl group stretching (C=O) unique to the carboxylic acid group in TPGS-COOH. The broad peak around 3300–3500 cm^−1^ further confirms the formation of TPGS-COOH by indicating O-H stretching in the carboxylic acid. Finally, the peak observed at 2930.67 cm^−1^ suggests C-H stretching in the alkyl group of TPGS-COOH.

#### 4.4.2. Characterization of iRGD-TPGS-COOH by FTIR

The FTIR spectra of iRGD peptide revealed characteristic peaks at 1436.46 cm^−1^, indicating a cyclic peptide group, and at 1198.92 cm^−1^, attributed to C-N stretching vibrations. Additionally, a broad peak observed between 2850 and 2950 cm^−1^ suggested C-H stretching vibrations in the aliphatic side chains of the peptide. After conjugation of iRGD with TPGS-COOH, a shift in peaks was observed. The iRGD-TPGS-COOH conjugate exhibited peaks at 1746.32 cm^−1^, 1426.75 cm^−1^, 1132.02 cm^−1^, and sharp peaks around 2850–2950 cm^−1^, as shown in Figure 4b. The peaks observed at 1746.32 cm^−1^ and 1426.75 cm^−1^ confirmed the presence of the carbonyl group (C=O) of carboxylic acid from TPGS-COOH and the cyclic peptide group of iRGD, respectively. These peaks confirmed the successful conjugation of iRGD with TPGS-COOH. Additionally, the 1132.02 cm^−1^ peak suggested a shift in the C-N stretching group compared to the iRGD peptide alone.

#### 4.4.3. Characterization of Trast-TPGS-COOH by FTIR

The FTIR profile of Trast revealed main peaks at around 1700 cm^−1^, 1500 cm^−1^, and 2800–2900 cm^−1^, as observed in Figure 4c. The peak at 1700 cm^−1^ (Amide I band, C=O stretching vibration) indicates a predominantly antiparallel beta-sheet structure typical of antibodies [77]. The peaks at around 1500 cm^−1^ correspond to the Amide II band, associated with N-H bending vibrations. Conjugation of Trast with TPGS-COOH was confirmed by the peaks observed at around 1745 cm^−1^, 880 cm^−1^, and 2928 cm^−1^. The weak signal around 1745 cm cm^−1^ indicates successful conjugation of Trast with TPGS-COOH. The decrease in the intensity of the 880 cm cm^−1^ peak (C-H bending vibrations) in the Trast-TPGS-COOH group signified successful conjugation [77].

#### 4.4.4. Characterization of DSDL Formulation

**Figure 5:**
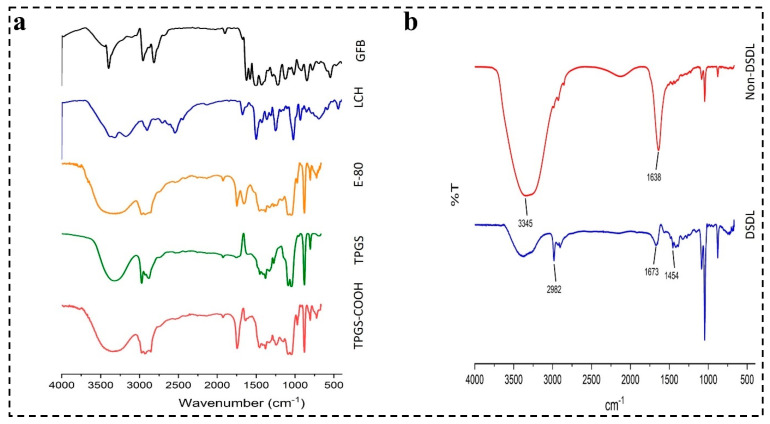
FTIR spectra of (**a**) TPGS, TPGS-COOH, E-80, LCH, and GFB and (**b**) DSDL and non-DSDL formulations.

The FTIR spectra of GFB, LCH, E-80, CHOL, TPGS-COOH along with DSDL and non-DSDL formulations are shown in Figure 5a,b. The peaks observed at 1673 cm^−1^ indicate the -C=O stretching of iRGD and the peak at 1454 cm^−1^ showed the presence of a cyclic peptide present in the iRGD peptide, as depicted in Figure 5b. The characteristic peaks at 3367 cm^−1^ and 2982 cm^−1^ indicate the presence of an -OH group and an overlapping of -CH stretching. The -CH bending is observed at 878 cm^−1^. The peaks observed in the DSDL spectra indicated the successful surface decoration of iRGD and Trast on liposome formulation.

### 4.5. EE (%) and DL (%)

**Figure 6:**
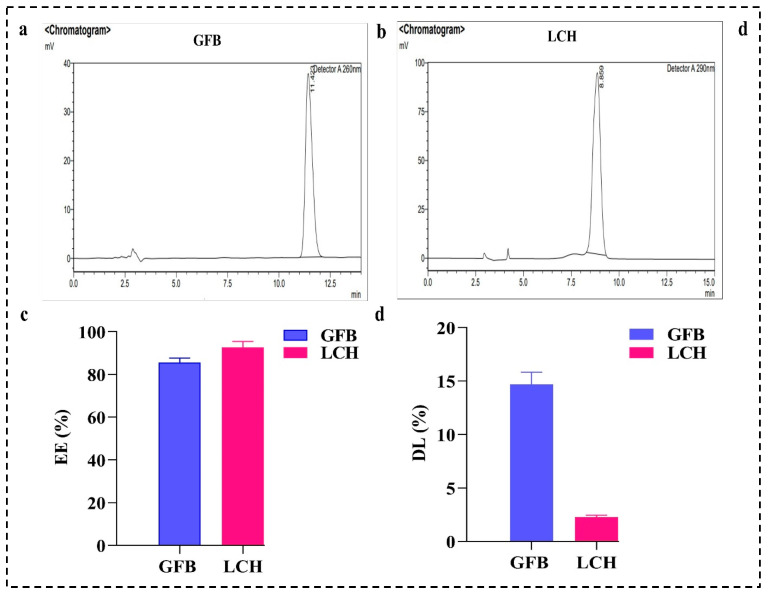
(**a**) RP-HPLC chromatogram of GFB and (**b**) LCH developed using a C18 column at a flow rate of 1 mL/min, with detection at wavelengths of 254 nm and 290 nm, respectively. (**c**) EE (%) and (**d**) DL (%) of GFB and LCH calculated using RP-HPLC.

The EE (%) of both the GFB and LCH drugs was calculated with a nanosep tube using RP-HPLC. Figure 6a,b represents the chromatogram of GFB and LCH, respectively. The observed EE (%) of the optimized SDLs was found to be 85.58 ± 2.11 and 92.62 ± 2.87% for GFB and LCH, respectively, as represented in Figure 6c,d. The percentage of DL of the DSDLs was also calculated and found to be of 14.68 ± 1.14% for GFB and 2.28 ± 0.18 for LCH, respectively, as shown in Figure 6d.

### 4.6. In-Vitro Drug Release Profile

**Figure 7:**
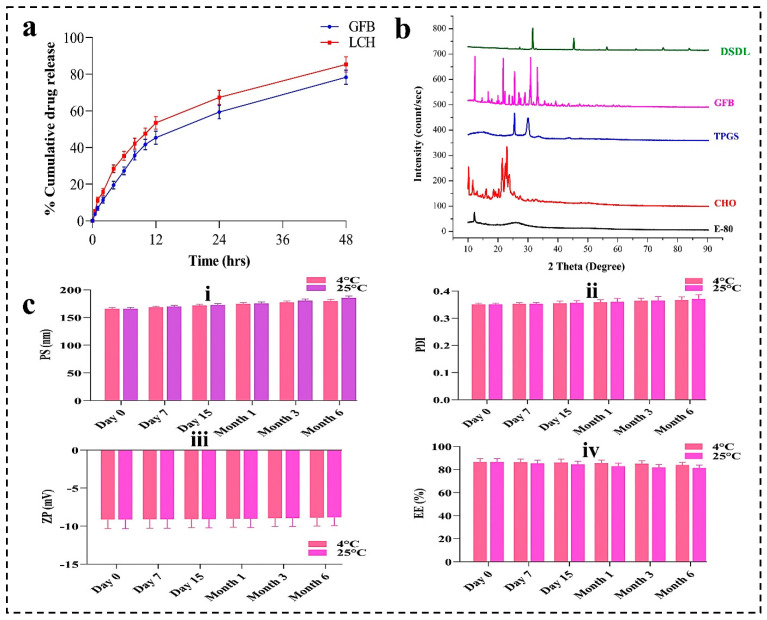
(**a**) Cumulative drug release (%) of GFB and LCH in PBS (pH 7.4) from DSDL formulation. (**b**) XRD spectra of E-80, cholesterol, TPGS, GFB, and DSDL formulation. (**c**) Stability study of DSDL formulation represented the change in PS, PDI, and ZP within 6 months.

The release kinetics of GFB and LCH are depicted in Figure 7a. After 12 h, the release percentages for GFB and LCH from DSDL formulations were 45.38 ± 3.56% and 53.46 ± 3.46%, respectively. Upon extending the incubation to 48 h, the cumulative release of GFB and LCH reached approximately 78.39 ± 3.87% and 85.37 ± 4.18%, respectively. The protracted and regulated release profile of GFB and LCH can be ascribed to prolonged systemic circulation times. This sustained release mechanism from DSDL formulations not only augments the targeting precision and therapeutic efficacy of anticancer agents but also mitigates systemic toxicity and reduces the emergence of drug resistance in cancer cells.

### 4.7. XRD Analysis

The XRD patterns of the various drugs and excipients are illustrated in Figure 7b. It is evident from the figure that E-80 exhibits an amorphous structure, in contrast to the other substances, which display crystalline to semicrystalline characteristics. Notably, the DSDL sample demonstrates a mixed phase, ranging from semicrystalline to amorphous. This indicates the successful encapsulation of drugs within the liposome architecture. The amorphous nature of DSDL formulations imparts unique properties, enhancing their functional performance.

### 4.8. Stability Studies

A stability study was performed for DSDL formulation at 25 °C ± 2 °C/60 ± 5% relative humidity (RH), as per the ICH guidelines and as shown in Figure 7c. Sampling was made at 0 days, 7 days, 15 days, 1 month, 3 months, and 6 month and characterized for PS, PDI, and ZP. The results are shown in Figure 7c. After analyzing, it may be concluded that there is not much more difference in the PS, PDI, and ZP of the formulations after 6 months. These results suggest that DSDLs are stable for up to 6 months.

### 4.9. Biocompatibility Study

**Figure 8:**
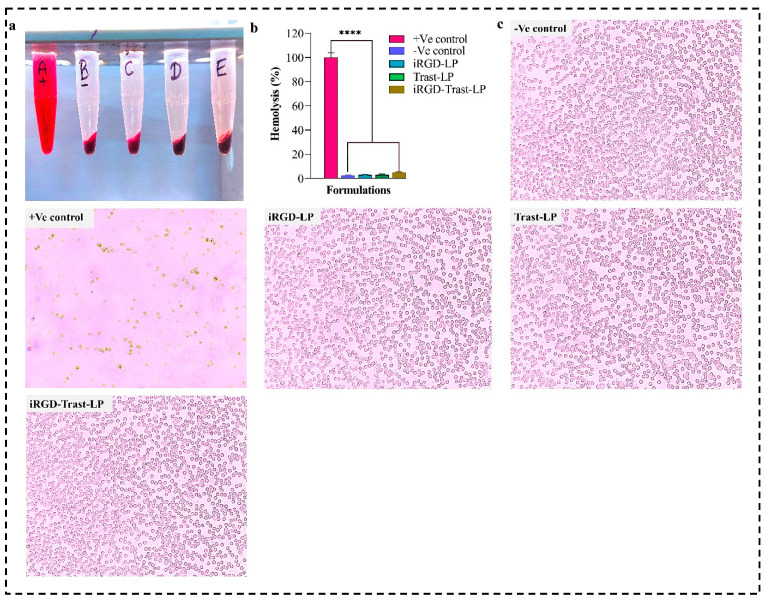
(**a**) Hemolysis assay of +Ve control (A), -Ve control (B), iRGD-LP (C), Trast-LP (D), and iRGD-Trast-LP (E). (**b**) Graph representing the % hemolysis of all the groups. (**c**) Microscopic images of RBCs of SDL and DSDL groups after hemolysis. The images were captured at 20× magnification. All the data represented as mean ± SD, (*n* = 3). *p* < 0.0001, denoted by **** and indicates highly significant data.

A hemolysis study was mainly employed to assess the potential toxicity of the prepared SDLs and DSDLs on RBCs. As shown in Figure 8a,b, SDL (iRGD-LP and Trast-LP) formulations demonstrated minimal hemolytic activity, with hemolysis percentages ranging from 2.94 ± 0.37% to 3.08 ± 0.48% for concentrations equivalent to 50 μg/mL. These low hemolysis values indicate that the liposomal formulations maintain RBCs’ integrity and do not induce significant lysis, suggesting good biocompatibility. The highest hemolytic activity was observed in the DSDL (iRGD-Trast-LP) group around 4.82 ± 0.57%, which also falls under the 5%. Hemolytic activity above 5% would raise safety concerns for intravenous administration or other applications involving blood interactions. Furthermore, the microscopic images of all the groups are shown in Figure 8c. Numerous studies have documented the in-vitro and in vivo biocompatibility of phospholipid-based liposomes, and these formulations have been explored as drug carriers due to their ability to minimize hemolysis and reduce side effects.

### 4.10. In-Vitro Cell Line

#### 4.10.1. Cytotoxicity Studies

**Figure 9:**
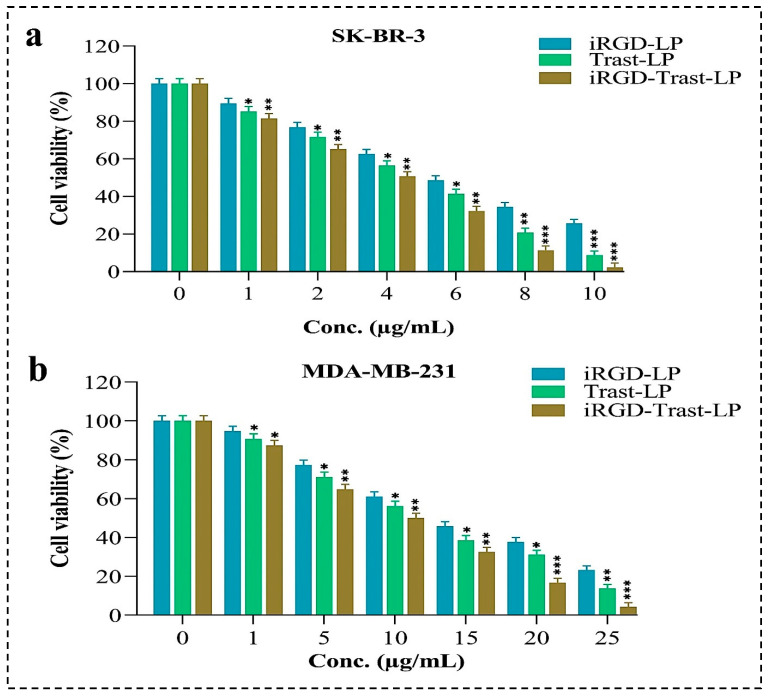
In-vitro cytotoxicity study of different SDL and DSDL formulations to calculate the IC-50 value on (**a**) SK-BR-3 and (**b**) MDA-MB-231 breast cancers cells. All the data represented as mean ± SD, (n = 3). *p* < 0.05, 0.01, and 0.001 are denoted by *, **, and ***, respectively. * Represents that the data are significant whereas ** and *** indicate highly significant data.

Cytotoxicity studies were conducted on SDLs and DSDLs at concentrations ranging from 1 to 10 μg/mL for SK-BR-3 and from 1 to 25 μg/mL for MDA-MB-231 to determine the IC-50 value and the impact of SDLs and DSDLs on these cell lines. The results of the MTT test can be seen in Figure 9. Based on the results, SDL and DSDL formulations had a substantial effect on the growth of these cell lines. There was a linear relationship between the concentrations of SDLs (iRGD-LP, Trast-LP), as well as DSDLs (iRGD-Trast-LP) and their growth inhibition. The IC-50 values of iRGD-LP, Trast-LP, and iRGD-Trast-LP against SK-BR-3 cells were found to be 6.10 ± 0.35, 4.98 ± 0.32, and 4.34 ± 0.28 μg/mL, respectively, as shown in Figure 9a. Similarly, the IC-50 values of iRGD-LP, Trast-LP, and iRGD-Trast-LP were found to be 15.12 ± 1.27, 13.09 ± 1.08, and 11.08 ± 0.74 μg/mL, respectively, for MDA-MB-23, indicating that DSDLs had significantly greater antitumor activity than SDLs, as depicted in Figure 9b. More importantly, DSDLs exhibited much higher anticancer activity for SK-BR-3, as evidenced by the low IC-50 value in Figure 9a.

#### 4.10.2. Cellular Uptake of SDLs and DSDLs on SK-BR-3 and MDA-MB-231

**Figure 10:**
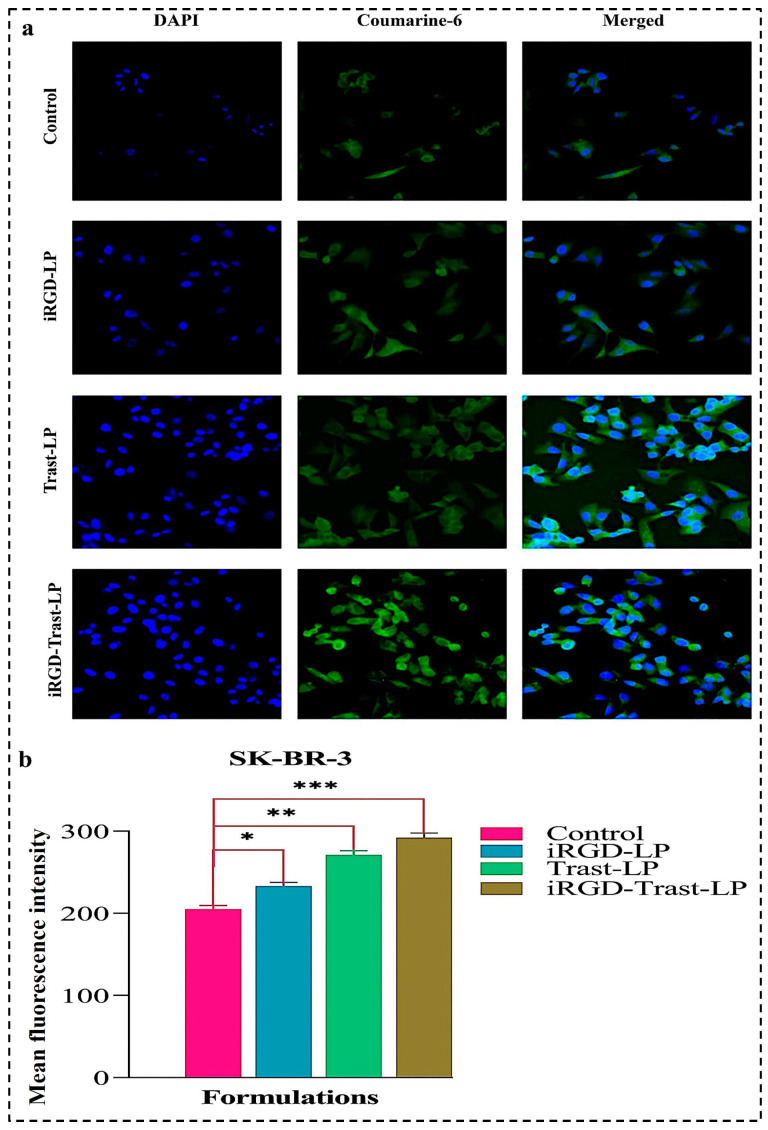
(**a**) Cellular internalization of control, iRGD-LP, Trast-LP, and iRGD-Trast-LP on SK-BR-3 cells. The images were captured at a magnification of 20. (**b**) Quantification of cellular uptake was performed by measuring MFI value using ImageJ software version 1.54g. All data are presented as mean ± SD with *n* = 3. * denotes *p* < 0.05, considered significant; ** and *** denote *p* < 0.01 and *p* < 0.001, respectively, considered highly significant data.

Coumarin-6-loaded placebo SDL (iRGD-LP and Trast-LP) and DSDL formulations were used for a cellular uptake study. The control (conventional placebo liposome), iRGD-LP, Trast-LP, and iRGD-Trast-LP were incubated with SK-BR-3 cells seeded in a 12-well plate on coverslips. Figure 10a shows the uptake of all the formulations, confirming that surface decoration significantly increased cellular uptake compared to the control group. The mean fluorescence intensity (MFI) observed in Figure 10b evidences that cellular internalization was significantly higher in the iRGD-LP, Trast-LP, and iRGD-Trast-LP groups compared to the control group.

**Figure 11:**
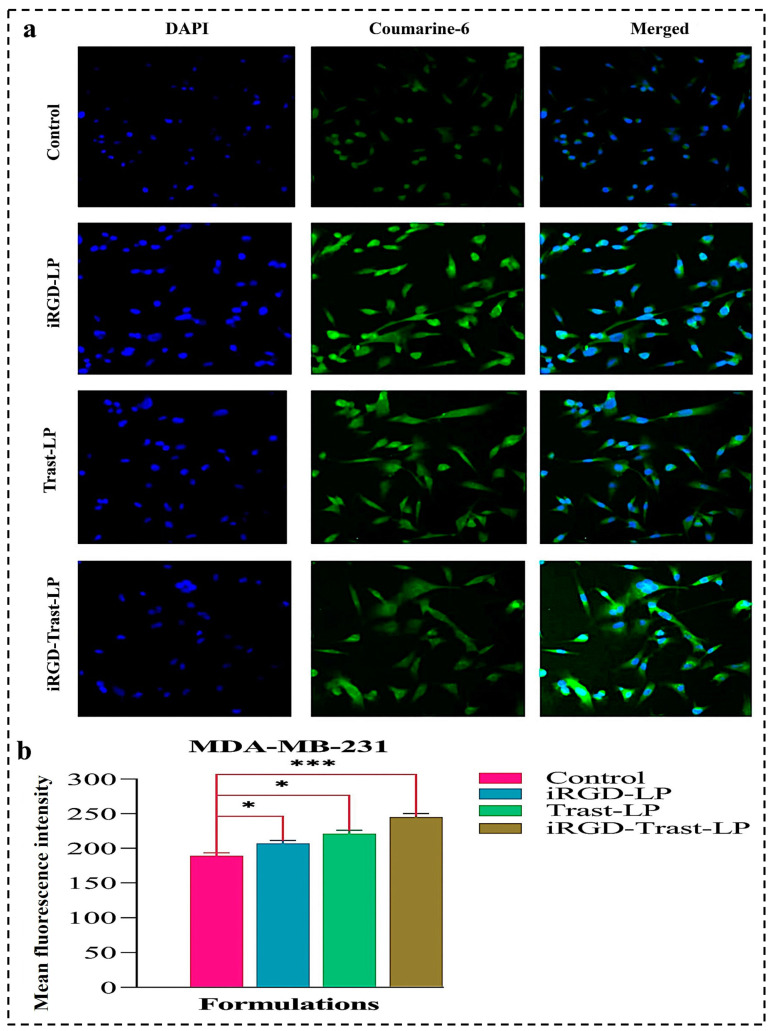
(**a**) Cellular uptake of control, iRGD-LP, Trast-LP, and iRGD-Trast-LP on MDA-MB-231 cells. All the images were photographed at a magnification of 20×. (**b**) Quantification of cellular internalization was performed by measuring mean MFI value using ImageJ software version 1.54g. All data are presented as mean ± SD with *n* = 3. * denotes *p* < 0.05, considered significant; and *** denote *p* < 0.001 considered highly significant data.

Furthermore, the cellular uptakes of Coumarine-6-loaded control, SDLs, and DSDLs were also performed on MDA-MB-231 cells, as shown in Figure 11a. The MFI value of cellular uptake of the control, iRGD-LP, Trast-LP, and iRGD-Trast-LP groups on MDA-MB-231 cells were measured using ImageJ software. The quantitative analysis of uptake is represented in Figure 11b and confirmed that surface decoration significantly improved the cellular uptake of SDL as well as DSDL formulations as compared to the control. It may be concluded from Figure 11b that cellular internalization was more present in the iRGD-LP, Trast-LP, and iRGD-Trast-LP groups than in the control group.

#### 4.10.3. Wound-Healing Assay

**Figure 12:**
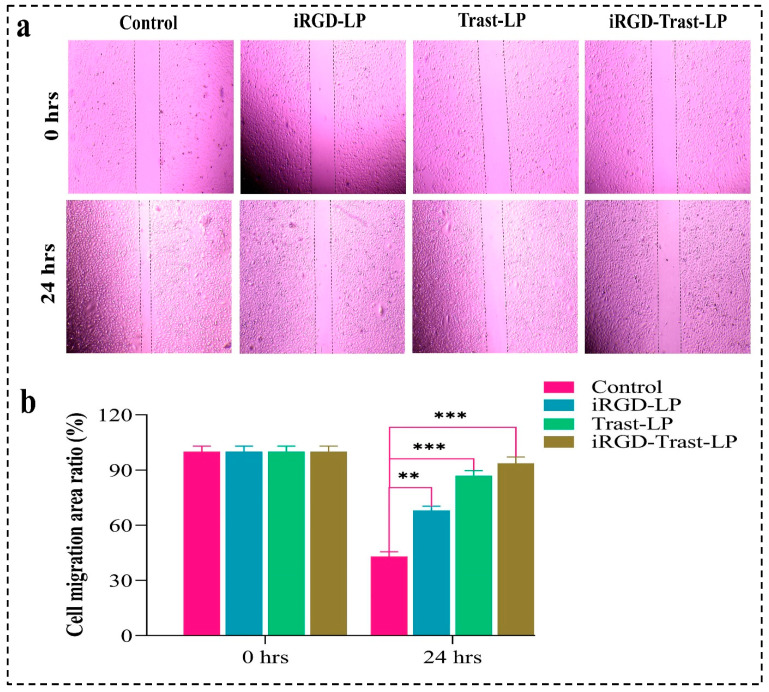
(**a**) Microscopic images of wound-healing assay of control, iRGD-LP, Trast-LP, and iRGD-Trast-LP. (**b**) Wound-healing area ratio (%) calculated by imageJ software version 1.54g. All the images were captured at a magnification of 10×. All the data represented as mean ± SD (*n* = 3). *p* < 0.01 was considered as highly significant and shown by **. *p* < 0.001 was represented by *** and also considered as highly significant.

Cell migration of MDA-MB-231 cells was tested to assess the effect of SDL and DSDL formulations. A wound-healing assay was performed to confirm the potential inhibitory effect of these formulations on MDA-MB-231 cell migration. Figure 12a clearly demonstrates that SDL and DSDL formulations have a potential inhibitory effect on the migration of MDA-MB-231 cells compared to control groups. Figure 12b shows the wound-healing ratio (%) of all the formulations. As expected, the control group exhibited the highest cell migration, while iRGD-Trast-LP showed the lowest. The control group had the lowest wound-healing percentage ratio (43.05 ± 2.64%), whereas iRGD-LP, Trast-LP, and iRGD-Trast-LP showed significantly higher ratios (*p* < 0.001). These findings suggest that SDL and DSDL formulations significantly impact the reduction in cell migration and motility of MDA-MB-231 cells and significantly reduce their wound-healing capability.

## 5. Discussion

Liposomes based on TPGS-COOH encapsulated with GFB and LCH anticancer drugs were successfully developed by the solvent injection method and optimized using the BBD approach in Design Expert software. The optimized liposomes were further surface-functionalized with cyclic peptide (iRGD) and monoclonal antibody Trast to enhance their targeting efficacy and penetration potential within tumor cells. TPGS was successfully activated to TPGS-COOH by a ring-opening polymerization mechanism. Trast and iRGD were successfully conjugated with TPGS-COOH to form the Trast-TPGS-COOH and iRGD-TPGS-COOH conjugates, respectively. FTIR analysis confirmed the activation of TPGS-COOH from TPGS and the conjugation of TPGS-COOH with Trast and iRGD. PS, PDI, and ZP analysis showed that the DSDL formulation was nanorange in size with a PS of 165.9 nm and a PDI value of 0.351, indicating that it was uniformly distributed within the system, as well as a ZP value of −9.12 mV, which suggests a highly stable liposome. However, a PDI value below 0.5 is considered to be indicative of a homogeneous distribution in a nanoformulation. A further reduction in PDI value (PDI < 0.3) demonstrates an excellent uniform distribution of liposomes within the formulation. The observed PDI value of 0.351 falls within the acceptable range of 0.5. The slightly higher PDI value may be attributed to various factors involved in the production of liposomes, such as lipid content, sonication, and the loading of two different anticancer drugs. Similarly, the ZP value of −9.12 mV indicates the high stability of the DSDL formulation. Additionally, a more negatively charged ZP represents the exceptional stability of the liposome formulation. Furthermore, FTIR spectra of DSDL and non-DSDL formulations demonstrated the successful surface decoration of Trast and iRGD on these formulations. XRD studies revealed the semicrystalline-to-amorphous nature of the DSDL formulation and the successful incorporation of GFB and LCH within the liposome structure. SEM and TEM images demonstrated that the DSDL liposomes were spherical and ranged between 150 and 200 nm in size. Surface decoration with iRGD and Trast slightly increased the PS of the DSDL formulations.

The EE (%) of DSDLs was found to be 86.58 ± 3.11% for GFB and 90.74 ± 4.76% for LCH. The DL (%) for DSDLs was observed to be 11.68 ± 1.16% for GFB and 2.03 ± 0.18% for LCH. In-vitro cumulative drug release (%) exhibited prolonged and controlled release of GFB and LCH from the DSDL formulation. The sustained release of the DSDL formulation enhanced the therapeutic and targeting efficiency against breast cancer cells. The slow release of GFB and LCH at the target site not only enhanced therapeutic activity but also reduced drug resistance developed by breast cancer cells. Combination therapy offers many advantages over other therapies, such as reduced drug resistance, synergistic effects, and reduced toxicity. A multidrug carrier can deliver multiple therapeutic agents simultaneously, potentially improving the therapeutic efficacy of different drugs with the complementary mechanism of action. The stability study of the DSDL formulation over 6 months exhibited slight changes in PS, PDI, and ZP, indicating excellent stability. The biological safety of any nanoformulation is crucial and must be assessed. To evaluate the biocompatibility of the developed SDLs and DSDLs, a hemolysis assay was performed on rat blood. The microscopic images of the treated SDL and DSDL formulations as well as those of the negative control groups indicated no significant lysis observed. Thus, the developed nanoformulation is safe and biocompatible. Additionally, the anticancer activity of SDL and DSDL formulations on breast cancer cell lines (SK-BR-3 and MDA-MB-231) demonstrated that dual surface decoration significantly enhanced anticancer activity.

Moreover, the DSDL formulation demonstrated greater anticancer activity against SK-BR-3 compared to MDA-MB-231 as compared to the SDL formulation, as evidenced by their IC-50 values. Furthermore, the cellular uptake of these SDL and DSDL formulations were also assessed on SK-BR-3 and MDA-MB-231 breast cancer cell lines. Cellular uptake results conclude that the DSDL formulation exhibited significantly higher cellular internalization potential compared to SDL formulations. The cellular internalization was further quantitatively assessed by measuring the mean fluorescence intensity (MFI). The MFI results, as evidenced from Figure 10b, demonstrate that dual decoration significantly enhanced cellular uptake in SK-BR-3 cells. Similar results were observed for MDA-MB-231 cells, as evidenced from Figure 11b. The MFI results clearly indicate that cellular uptake in single-functionalized liposomal formulations was significantly lower compared to dual-functionalized liposomes. Therefore, it may be concluded that iRGD and Trast DSDL may be important therapeutic tools for metastatic breast cancer cells.

Furthermore, the cell migration potentials of these developed SDL and DSDL formulations were evaluated on MDA-MB-231 cells using a wound-healing assay. The cell migration of SDL-treated, DSDL-treated, and control groups after 24 h was measured and represented as a wound-healing area ratio (%). The wound-healing assay results demonstrate that SDL and DSDL treatment significantly decreased the cell migration of MDA-MB-231 cells after 24 h compared to the control group. Additionally, the DSDL formulation exhibited a significantly lower cell migration of MDA-MB-231 cells compared to SDL formulations after 24 h. From the above results, it can be concluded that iRGD and Trast functionalization leads to better cytotoxic effects on SK-BR-3 and MDA-MB-231 cells and may be a promising candidate for HER2-positive metastatic breast cancer treatment.

## 6. Conclusions

GFB and LCH dual-drug loaded TPGS-COOH-based liposomes were successfully developed, optimized, and surface-functionalized with iRGD and Trast. The FTIR results confirm the successful activation of TPGS-COOH and decoration of iRGD and Trast on the surface of the liposomes. The in-vitro results demonstrate that the prepared liposomes were spherical and within the nanoscale range of 200 nm. FTIR and XRD data revealed the successful incorporation of GFB and LCH within DSDLs, surface functionalization, and the transition from semi-crystalline to amorphous nature. The biological compatibility and biosafety of SDLs and DSDLs were assessed via hemolytic analysis, showing no significant lysis in the SDL and DSDL-treated groups. Additionally, in-vitro cell line studies revealed significant cytotoxicity, cellular internalization, and reduction in cell migration in SK-BR-3 and MDA-MB-231 breast cancer cells. The overall findings suggest that dual-drug-enriched, dual-surface-modified, multifunctional liposomes may be a promising therapeutic strategy for the treatment of HER2-positive metastatic breast cancer.

## Figures and Tables

**Table 1:** Different levels of independent variables (3 factors and 3 levels) by using BBD for optimization of TPGS-based liposomes.

Variables	Levels		
	−1	0	+1
Independent variables			
X1 = E-80 (mg)	30	45	60
X2 = Cholesterol (mg)	10	15	20
X3 = TPGS (mg)	5	7.5	10
Dependent variables	Desired response		
Y1 = PS (nm)	Minimum		
Y2 = PDI	Minimum Maximum		
Y3 = ZP (±)			

**Table 2:** Total number of formulations generated by BBD representing the value of independent variables (E-80, cholesterol, and TPGS) and their corresponding values of dependent variables (PS, PDI, and ZP).

Independent Variable (Factors)				Dependent Variable (Response)		
Formulation Code	Factor 1 (X1)	Factor 2 (X2)	Factor 3 (X3)	PS (nm) Y1	PDI Y2	ZP (±mV) Y3
LP1	45	15	7.5	152.8 ± 1.54	0.375 ± 0.046	−7.36
LP 2	60	15	5	189.4 ± 2.06	0.423 ± 0.068	−11.32
LP 3	30	15	5	135.5 ± 1.36	0.355 ± 0.034	−9.1
LP 4	30	15	10	143.5 ± 1.48	0.362 ± 0.081	−6.31
LP 5	30	20	7.5	149.2 ± 1.59	0.348 ± 0.059	−8.26
LP 6	45	20	5	164.3 ± 1.85	0.318 ± 0.047	−9.69
LP 7	60	15	10	194.6 ± 2.18	0.463 ± 0.065	−9.2
LP 8	60	10	7.5	180.6 ± 2.03	0.494 ± 0.072	−10.3
LP 9	45	10	5	147.9 ± 1.47	0.395 ± 0.061	−8.19
LP 10	45	15	7.5	152.8 ± 1.54	0.375 ± 0.058	−7.669
LP 11	30	10	7.5	114.7 ± 1.16	0.385 ± 0.055	−8.88
LP 12	45	10	10	151.1 ± 1.54	0.378 ± 0.053	−6.8
LP 13	45	15	7.5	152.8 ± 1.54	0.375 ± 0.062	−7.36
LP 14	45	20	10	183.6 ± 1.97	0.325 ± 0.048	−9.16
LP 15	60	20	7.5	198.4 ± 2.38	0.338 ± 0.056	−15.62

## Data Availability

All datasets that support the study are available from the corresponding author upon reasonable request.

## Abbreviations

HER-2	Human epidermal growth factor receptor-2
Trast	Trastuzumab
GFB	Gefitinib
LCH	Lycorine hydrochloride
TPGS-COOH	Activated D-Alpha-Tocopheryl polyethylene glycol 1000 succinate
DSDL	Dual surface-decorated liposomes
PS	Particle size
PDI	Polydispersity index
ZP	Zeta potential
IC-50	Half-maximum inhibitory concentration
SDL	Single decorated liposomes
CLSM	Confocal laser scanning microscopy
ErbB-2	Erythroblastic leukemia viral oncogene homolog-2
RGDK	Cryptic CendR motif
NRP-1	Neuropilin-1
TCRP1	Tongue cancer resistance-associated protein
Akt	Protein kinase B
mTOR	Mammalian target of rapamycin
BCS	Biopharmaceutical classification system
MAPK/PI3K	Mitogen-activated protein kinase/phosphatidylinositol 3-kinase
TME	Tumor microenvironment
TPGS	D-Alpha-Tocopheryl polyethylene glycol 1000 succinate
HLB	Hydrophilic and lipophilic balance
FDA	Food and drug administration
DSPE-PEG2000	1,2-distearoyl-sn-glycero-3-phosphoethanolamine-N-[amino(polyethylene glycol)-2000]
CAFs	Cancer-associated fibroblasts
ECM	Extracellular matrix
TAMs	Tumor-associated macrophages
MDSCs	Myeloid-derived suppressor cells
TGF-β	Transforming growth factor beta
VEGF	Vascular endothelial growth factor
TILs	Tumor-infiltrating lymphocytes
HIFs	Hypoxia-inducible factors
PD-L1	Programmed-death ligand
CTLA-4	Cytotoxic T-lymphocyte-associated protein-4
NKG-2A	Natural killer group 2A
EDC	1-Ethyl-3-[3-dimethylaminopropyl] carbodiimide hydrochloride
NHS	N-hydroxy succinimide
DMAP	4-(Dimethyl amino) pyridine
MTT	3-(4,5-dimethylthiazol-2-yl)-2,5-diphenyltetrazolium bromide
DAPI	4′,6-diamidino-2-phenylindole dihydrochloride
DMEM	Dulbecco’s Modified Eagle Medium
FBS	Fetal bovine serum
DCM	Dichloromethane
PBS	Phosphate buffered saline
RSM	Response surface methodology
DOE	Design of experiments
BBD	Box–Behnken design
R^2^	Correlation coefficient
RMSE	Root mean square error
SEM	Scanning electron microscopy
TEM	Transmission electron microscopy
XRD	X-ray diffraction
FTIR	Fourier transform infrared spectroscopy
EE	Entrapment efficiency
DL	Drug loading
RP-HPLC	Reverse-phase high-performance liquid chromatography
ICH	International Council for Harmonization of Technical Requirements for Pharmaceuticals for Human Use
ACN	Acetonitrile
TFA	Trifluoroacetic acid
NCCS	National Centre for Cell Science
DMSO	Dimethyl sulfoxide
CLSM	Confocal laser scanning microscopy
MFI	Mean fluorescence intensity
